# Increased TG to HDL-C ratio is associated with severity of drug-induced liver injury

**DOI:** 10.1038/s41598-023-34137-4

**Published:** 2023-04-27

**Authors:** Xiaoqing Jia, Xiaoting Zhang, Ming Yan, Dalong Sun, Rong Li, Na Yang, Zheng Luo

**Affiliations:** 1 Department of Gastroenterology, Qilu Hospital, Shandong University, 107 West Wenhua Road, Jinan, Shandong 250012 People’s Republic of China; 2 Department of Geriatric Medicine, Qilu Hospital, Shandong University, 107 West Wenhua Road, Jinan, Shandong 250012 People’s Republic of China

**Keywords:** Diseases, Gastroenterology, Risk factors

## Abstract

We investigated the relationship between dyslipidemia and drug-induced liver injury (DILI), especially the level of triglyceride to high-density lipoprotein cholesterol ratio (TG/HDL-C) in severe DILI. In this single-centered retrospective study, of 326 patients with DILI, 221 patients were analyzed. Control groups include medication using group and acute hepatitis B group. The relationship between dyslipidemia and DILI was estimated. Demographic and clinical features were analyzed. Dyslipidemia and TG/HDL-C ratios were compared between DILI and control groups, DILI mild group and severe group. The area under the receiver-operating characteristic curve (AUC) was used to evaluate the credibility of the relationship and to find cut-off points. Dyslipidemia is related to DILI when compared with medication using control group (AOR 4.60; 95% CI 2.81–7.54; P < 0.01) and compared with acute hepatitis B group (AOR 2.12; 95% CI 1.37–3.29; P < 0.01). Dyslipidemia is associated with the severity of DILI (AOR 25.78; 95% CI 7.63–87.1; P < 0.01). TG/HDL-C ratio is higher in DILI group than that of medication using control group, also higher in severe DILI group than that of mild DILI group. AUCs for TG/HDL-C ratio to indicate the severity of DILI was 0.89 (P < 0.05), the cut-off point was 2.35. Dyslipidemia and TG/HDL-C ratio were related to DILI occurrence. Severe liver injury in DILI was associated with dyslipidemia and elevated TG/HDL-C ratio.

## Introduction

Drug-induced liver injury (DILI) is the most common causation of acute liver failure in the West^1^. In some Asian countries, patients with herb-induced liver injury (HILI) caused by traditional Chinese medicines have been analyzed in some medical reviews^2,3^. DILI is caused by adverse reactions to a medicine or drug combination. Risk factors for DILI are associated with metabolic, immunological, and genetic factors of the individuals^4,5^. Individuals who are aged, female, or have chronic liver injuries are more likely to suffer from DILI^6^. Recent studies found that dyslipidemia is a risk factor for DILI, and the relation between dyslipidemia and DILI has been studied^7^.

Dyslipidemia is a common, multifactorial disease that is a major determinant of the cardiovascular risk. Dyslipidemia includes high level of triglyceride (TG), cholesterol (Cho) and low-density lipoprotein (LDL-C), and low level of high-density lipoprotein cholesterol (HDL-C). The TG to HDL-C (TG/HDL-C) ratio summarizes some serum lipid levels, which has been confirmed to be a valuable predictor for cardio-metabolic risk, insulin resistance, cardiovascular diseases and the incidence of chronic kidney diseases^8–11^. Dyslipidemia and TG/HDL-C ratio has been reported to be associated with NAFLD^12,13^. Free fatty acid-induced lipotoxicity is an important feature of non-alcoholic fatty hepatitis pathogenesis^14^. Dyslipidemia has been estimated to be a risk factor for DILI^7^.

Few studies have been designed to investigate the relationship between dyslipidemia and severity of DILI to date. This study was performed to explore dyslipidemia and the level of TG/HDL-C ratio in DILI in China.

## Results

### Characteristics of DILI and control group members

**Figure 1:**
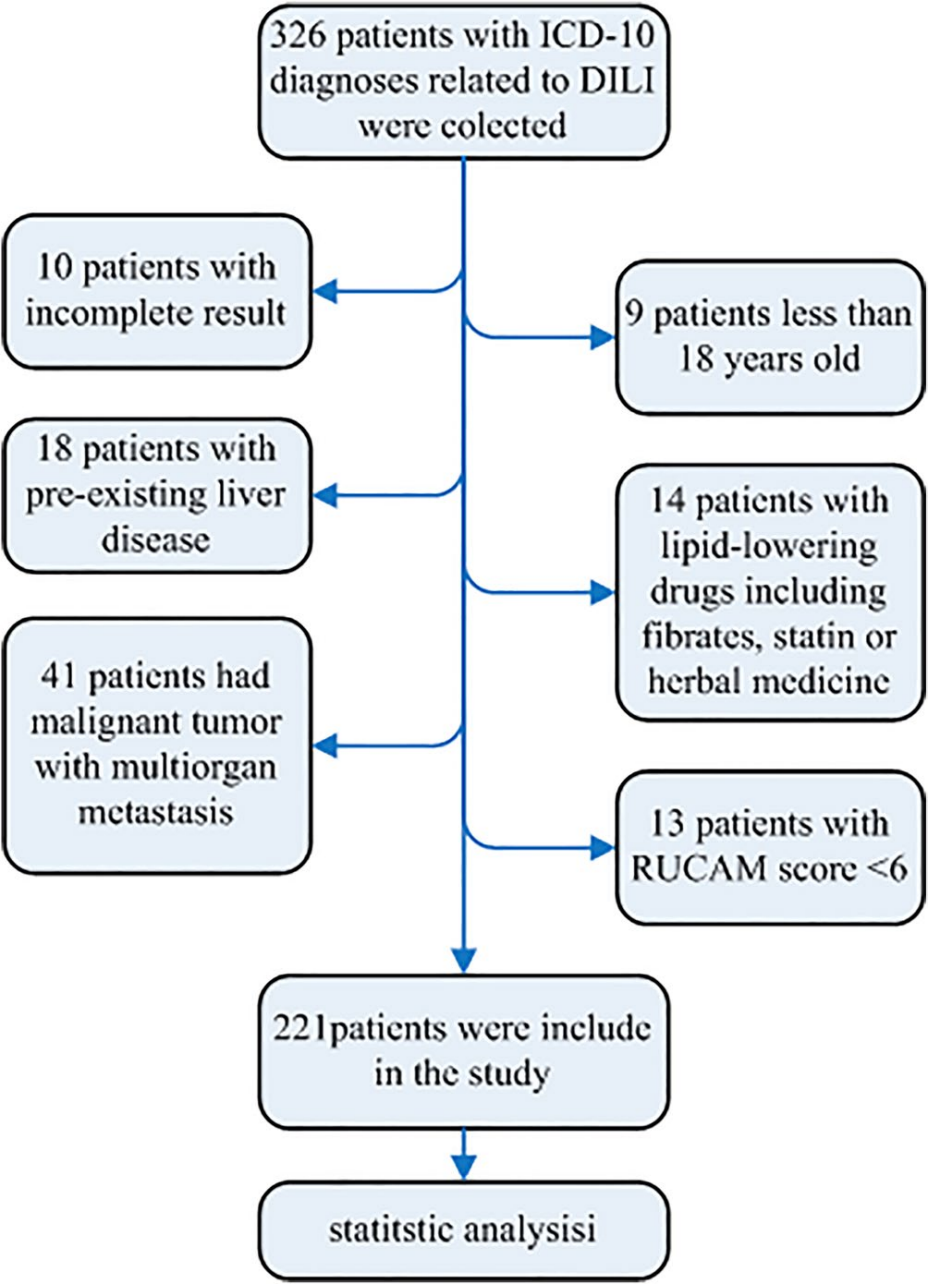
Flow chart of the study.

326 patients with DILI were assessed and 105 patients were excluded for a variety of reasons (Fig. 1). Demographic and clinical characteristics of 221 DILI patients, 246 medication using controls and 261 acute hepatitis B participants are shown in Table 1. Among DILI patients, 42.08% were males and 57.92% were females. The average age of the DILI group was 50.97. In the control group, 55.69% were males and 44.31% were females, and their average age was 55.15. In the acute hepatitis B group, 78.16% were males and 21.84% were females, with an average age of 43.17.

**Table 1:** Univariate analysis and multivariate analysis of patients’ characters in DILI and control groups.

Variable	DILI	Control				HBV			
			p1	AOR (95%CI) *	p2		p1	AOR (95%CI) **	p2
Sex			< 0.01	0.55 (0.34–0.9)	0.02		< 0.01	0.21 (0.12–0.35)	< 0.01
Female (N%)	57.92	44.31				21.84			
Male (N%)	42.08	55.69				78.16			
Age (mean ± SD)	50.97 ± 13.34	55.15 ± 14.92	< 0.01	0.67 (0.39–1.12)	0.13	43.17 ± 12.06	< 0.01	2.96 (1.69–5.18)	< 0.01
< 60	71.49	58.54				90.04			
≥ 60	28.51	41.46				9.96			
Smoking			0.55	–	–		< 0.01	0.89 (0.48–1.64)	0.70
No (N%)	75.57	73.17				59.39			
Yes (N%)	24.43	26.83				40.61			
Alcoholic abuse			0.97	–	–		< 0.01	0.97 (0.52–1.8)	0.92
No (N%)	74.66	74.80				56.32			
Yes (N%)	25.34	25.20				43.68			
Hypertension			< 0.01	0.49 (0.27–0.88)	0.02		< 0.01	2.48 (1.34–4.61)	< 0.01
No (N%)	80.09	60.16				90.42			
Yes (N%)	19.91	39.84				9.58			
Heart disease			0.05	0.63 (0.22–1.82)	0.40		0.06	1.1 (0.27–4.49)	0.89
No (N%)	95.48	90.65				98.47			
Yes (N%)	4.52	9.35				1.53			
Diabetes			< 0.01	0.41 (0.17–1.01)	0.05		0.16	–	–
No (N%)	94.57	84.55				91.19			
Yes (N%)	5.43	15.45				8.81			
Malignant disease			0.02	11.39 (4.96–26.15)	< 0.01		1.00	–	–
No (N%)	84.16	95.12				100.00			
Yes (N%)	15.84	4.88				0.00			
Allergic history			< 0.01	1.92 (1.01–3.65)	0.05		< 0.01	3.84 (1.98–7.43)	< 0.01
No (N%)	79.64	87.40				93.10			
Yes (N%)	20.36	12.60				6.90			
Family chronic disease			0.04	3.33 (1.23–9.04)	0.02		< 0.01	0.44 (0.23–0.84)	0.01
No (N%)	90.50	95.53				80.08			
Yes (N%)	9.50	4.47				19.92			
Dyslipidemia			< 0.01	4.6 (2.81–7.54)	< 0.01		0.01	2.12 (1.37–3.29)	< 0.01
No (N%)	36.20	67.48				48.66			
Yes (N%)	63.80	32.52				51.34			
Medication type			< 0.01	0.24 (0.18–0.33)	< 0.01				
Chinese herbal medicines	50.68	5.69							
Both	15.38	22.76							
Western medicines	33.94	71.54							

*DILI* drug induced liver injury, *AOR* adjusted odds ratio.

*p1* p value for univariate analysis, *p2* p value for multivariate analysis. *Adjusted for sex, age, hypertension, heart disease, diabetes, history of malignancy, allergic history, family chronic diseases, dyslipidemia and medication type. **Adjusted for sex, age, smoking, alcoholic abuse, hypertension, heart disease, allergic history, family chronic diseases and dyslipidemia.

According to Chinese Society of Hepatology Guidelines, the severity of DILI was defined in 5 levels^15^. To investigate the severity of DILI, patients were divided into two groups, mild (level 0–2, 148 patients) group and severe (≥ level 3, 73 patients) group. Of the mild group, 37.84% were males and 62.16% were females, with an average age of 50.84. Then in the severe group, 50.68% were males and 49.32% were females, and their average age was 51.23 (Table 2).

**Table 2:** Univariate analysis and multivariate analysis of patients’ characters in mild and severe DILI group.

Variable	Level 0–2	Level ≥ 3	P1	AOR (95% CI)*	P2
Sex			0.07	1.31 (0.56–3.07)	0.53
Female (N%)	62.16	49.32			
Male (N%)	37.84	50.68			
Age (mean ± SD)	50.84 ± 13.05	51.23 ± 14.01	0.57	–	–
< 60	70.27	73.97			
≥ 60	29.73	26.03			
Smoking			0.09	1.38 (0.53–3.58)	0.50
No (N%)	79.05	68.49			
Yes (N%)	20.95	31.51			
Alcoholic abuse			0.14	–	–
No (N%)	77.70	68.49			
Yes (N%)	22.30	31.51			
Hypertension			0.37	–	–
No (N%)	78.38	83.56			
Yes (N%)	21.62	16.44			
Heart disease			0.38	–	–
No (N%)	94.59	97.26			
Yes (N%)	5.41	2.74			
Diabetes			0.23	–	–
No (N%)	93.24	97.26			
Yes (n%)	6.76	2.74			
Malignant disease			0.01	0.32 (0.1–0.97)	0.04
No (N%)	79.73	93.15			
Yes (N%)	20.27	6.85			
Allergic history			0.51	–	–
No (N%)	78.38	82.19			
Yes (n%)	21.62	17.81			
Family chronic disease			0.14	–	–
No (N%)	92.57	86.30			
Yes (N%)	7.43	13.70			
Dyslipidemia			< 0.01	25.78 (7.63–87.1)	< 0.01
No (N%)	36.49	1.37			
Yes (N%)	63.51	98.63			
Medication type			0.02	0.65 (0.44–0.95)	0.03
Chinese herbal medicines	46.62	63.01			
Both	14.86	13.70			
Western medicines	38.51	23.29			

*DILI* drug induced liver injury, *AOR*: adjusted odds ratio.

*P1* P value for univariate analysis, *P2* P value for multivariate analysis. *Adjusted for sex, smoking, malignant disease, dyslipidemia and medication type.

Drug classes in the DILI group and control group were listed in Tables 1 and 3. In DILI group, 75 patients (33.94%) used western medicines, 112 patients (50.68%) used Chinese herbal medicines, and 34 patients (15.38%) used a combination of the two. In medication using control group, 176 patients (71.54%) used western medicines, 14 patients (5.69%) used Chinese herbal medicines, and 56 patients (22.76%) used a combination of the two. There is a significant difference between drug classes of DILI and control groups with P < 0.01. In mild DILI group, 46.62% patients used Chinese herbal medicines, 38.51% patients used western medicines, and 14.86% patients used a combination of the two. In severe DILI group, 63.01% patients used Chinese herbal medicines, 23.29% patients used western medicines, and 13.70% patients used a combination of the two (Table 2). Liver injury classes of DILI were also investigated, 86 patients (38.91%) mainly had hepatocellular injury, 104 patients (47.06%) mainly had cholestatic injury, and 31 patients (14.03%) had mixed injuries (Table 3). In DILI group, 177 patients (80.09%) had medication less than 31 days, 34 patients (15.38%) had medication over 31 days but less than 1 year, 10 patients (4.52%) had medication more than 1 year (Table 3). The drug indication for DILI and medication using groups were shown in Table 3, with healthcare drugs, analgesics and antipyretics and antineoplastic drugs being the top three drugs inducing DILI, there is a significant difference between drug indications for DILI and medication using groups with P < 0.01.

**Table 3:** Different classes of DILI and control group.

	DILI group	Control group	P
Classes of drugs			
Chinese herbal medicines N(%)	112 (50.68)	14 (5.69)	< 0.01
Western medicines N(%)	75 (33.94)	176 (71.54)	
Both N(%)	34 (15.38)	56 (22.76)	
Patterns of liver injury			
Hepatocellular N(%)	94 (42.53)		–
Cholestatic N(%)	104 (47.06)		
Mix N(%)	23 (10.41)		
Severity of liver injury			
Level 1 N(%)	103 (46.61)		–
Level 2 N(%)	45 (20.36)		
Level 3 N(%)	30 (13.57)		
Level 4 N(%)	43 (19.46)		
Duration of medication			
≤ 31 days N(%)	177 (80.09)		–
32–365 days N(%)	34 (15.38)		
> 1 year N(%)	10 (4.52)		
Indication of drugs			
Cardiovascular drugs N(%)	8 (3.62)	77 (31.3)	< 0.01
Psychiatry drugs N(%)	11 (4.98)	10 (4.07)	
Analgesic and antipyretic drugs N(%)	46 (20.81)	72 (29.27)	
Antineoplastic drugs N(%)	26 (11.76)	6 (2.44)	
Digestive system drugs N(%)	7 (3.17)	5 (2.03)	
Obstetric/gynecological drugs N(%)	2 (0.9)	1 (0.41)	
Rheumatism drugs N(%)	2 (0.9)	60 (24.39)	
Endocrine drugs N(%)	7 (3.17)	27 (10.98)	
Urinary system drugs N(%)	6 (2.71)	3 (1.22)	
Healthy care drugs N(%)	83 (37.56)	32 (13.01)	
Anti-inflammatory drugs N(%)	18 (8.14)	9 (3.66)	
Others N(%)	21 (9.5)	12 (4.88)	

*DILI* drug induced liver injury.

A record of all drugs prescribed for each DILI cases is kept.

### Clinical and demographic characteristics associated with DILI

Compared with medication using group, results from the univariate analysis indicated that sex, age, hypertension, heart disease, diabetes, history of malignancy, allergic history, family chronic diseases, dyslipidemia and medication type had significant values less than 0.1 between patients in DILI and control group. Sex, age, hypertension, heart disease, diabetes, history of malignancy, allergic history, family chronic diseases, dyslipidemia and medication type were included in the multivariate analysis. DILI patients were more likely to have dyslipidemia with an adjusted odds ratio (AOR) of 4.6 (95% CI 2.81–7.54; P < 0.01). Patients who had hypertension had an AOR of 0.49 (95% CI 0.27–0.88; P = 0.02) to DILI compared with participants without hypertension. Patients who had malignant diseases had an AOR of 11.39 (95% CI 4.96–26.15; P < 0.01) to DILI compared with participants without malignant diseases. Participants who had family histories of chronic diseases had an AOR of 3.33 (95% CI 1.23–9.04; P = 0.02) to DILI compared with those without family histories of chronic diseases. When compared between participants using different types of drugs, participants using western medicine had lower risk of DILI when compared to participants with Chinese herbal medicine or both (AOR 0.24; 95% CI 1.23–9.04, .18–0.33; P < 0.01). There was no statistic significant association between age, smoking, alcohol abuse, CVD hypersensitivity, diabetes, or allergic history, and DILI. Above data were shown in Table 1.

Compared with acute hepatitis B group, results from the univariate analysis indicated that sex, age, smoking, alcoholic abuse, hypertension, heart disease, allergic history, family chronic diseases and dyslipidemia had significant value less than 0.1 between patients in DILI and acute hepatitis B group. Sex, age, smoking, alcoholic abuse, hypertension, heart disease, allergic history, family chronic diseases and dyslipidemia were included in the multivariate analysis. DILI participants were more likely to have dyslipidemia with an AOR of 2.12 (95% CI 1.37–3.29; P < 0.01) to DILI. DILI participants who were 60 or older had an AOR of 2.96 (95% CI 1.69–5.18; P < 0.01) to DILI compared with participants less than 60. Patients who had hypertension had an AOR of 2.48 (95% CI 1.34–4.61; P < 0.01) to DILI compared with participants without hypertension. Patients with hypersensitivity had an AOR of 3.84 (95% CI 1.98–7.43, P < 0.01) to DILI compared with those without hypersensitivity. Patients with family history of chronic diseases had an AOR of 0.44 (95% CI 0.23–0.84, P = 0.01) to DILI compared with those without family chronic diseases. There was no statistic significant association between smoking, alcoholic abuse, CVD, diabetes or malignant disease and the DILI. Above data were shown in Table 1.

DILI patients with dyslipidemia had an AOR of 25.78 (95% CI 7.63–87.1; P < 0.01) to severe DILI compared with patients with normal plasma lipid levels. DILI patients with malignant diseases had lower risk to severe DILI with an AOR of 0.32 (95% CI 0.1–0.97; P = 0.04). When compared between participants using different type of drugs, participants using western medicine had lower risk of severe DILI when compared to participants with Chinese herbal medicine or both(AOR: 0.65; 95% CI 0.44–0.95; P = 0.03) (Table 2).

### Dyslipidemia and TG/HDL-C ratio associated with DILI

**Figure 2:**
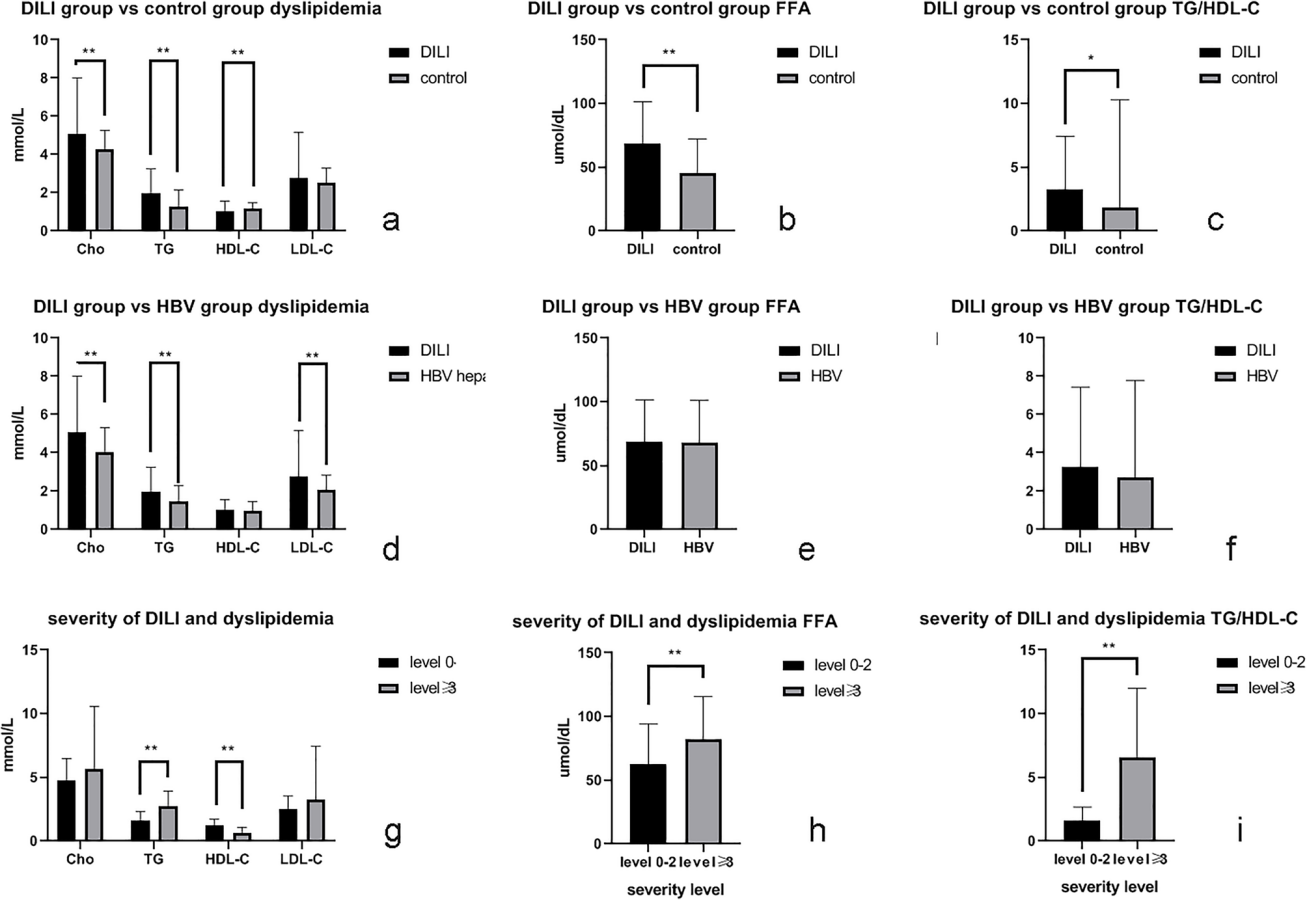
Dyslipidemia and TG/HDL-C ratio in DILI and control groups. *TG* triglyceride, *Cho* cholesterol, *LDL-C* low-density lipoprotein cholesterol, *HDL-C* high-density lipoprotein cholesterol, *FFA* free fatty acid, *DILI* drug induced liver injury. (**a**) Cho, TG, HDL-C and LDL-C levels in DILI and medication using control groups; (**b**) FFA level in DILI and medication using control groups; (**c**) TG/HDL-C ratio in DILI and medication using control groups; (**d**) Cho, TG, HDL-C and LDL-C levels in DILI and acute hepatitis B groups; (**e**) FFA level in DILI and acute hepatitis B groups; (**f**) TG/HDL-C ratio in DILI and acute hepatitis B groups; (**g**) Cho, TG, HDL-C and LDL-C levels in mild and severe DILI groups; (**h**) FFA level in mild and severe DILI groups; (**i**) TG/HDL-C ratio in mild and severe DILI groups.*P ≤ 0.05, **P ≤ 0.01.

Among all patients with DILI in this study, 75.11% had dyslipidemia (Table 1). Levels of Cho, TG, free fatty acid (FFA) and TG/HDL-C ratio were significantly higher in DILI groups than those in medication using control group. Levels of HDL-C were significantly lower in DILI groups than those in medication using control group (Table 4, Fig. 2). Levels of Cho, TG and LDL-C were significantly higher in DILI group than those in acute hepatitis B group. (Table 4, Fig. 2).

**Table 4:** Dyslipidemia and TG/HDL-C ratio in classes of DILI and control group.

Variable	Cho (mmol/L)		TG (mmol/L)		HDL-C (mmol/L)		LDL-C (mmol/L)		FFA (µmol/dL)		TG/HDL-C	
	(Mean ± SD)	P	(Mean ± SD)	P	(Mean ± SD)	P	(Mean ± SD)	P	(Mean ± SD)	P	(Mean ± SD)	P
DILI	5.07 ± 2.93		1.99 ± 1.26		1.03 ± 0.53		2.79 ± 2.37		68.98 ± 32.48		3.26 ± 4.17	
Control	4.28 ± 0.99	< 0.01	1.29 ± 0.85	< 0.01	1.15 ± 0.33	< 0.01	2.54 ± 0.75	0.14	45.64 ± 26.52	< 0.01	1.8 ± 8.51	0.02
HBV	4.04 ± 1.27	< 0.01	1.47 ± 0.82	< 0.01	0.99 ± 0.48	0.34	2.08 ± 0.76	< 0.01	68.08 ± 33	0.76	2.73 ± 5.05	0.22
Classes of drugs												
Chinese herbal medicines	4.83 ± 3.3	> 0.05	2.01 ± 1.11	> 0.05	0.94 ± 0.52^b^		2.57 ± 2.67	> 0.05	72.29 ± 32.69	> 0.05	3.75 ± 4.42^a^	
Western medicines	5.49 ± 2.55		1.79 ± 1.42		1.19 ± 0.5^a^		3.1 ± 1.96		63.81 ± 32.16		2.38 ± 3.85^b^	
Both	4.94 ± 2.26		2.35 ± 1.32		1 ± 0.58^ab^		2.83 ± 2.06		69.06 ± 31.9		3.53 ± 3.72^ab^	
Patterns of liver injury												
Hepatocellular	4.31 ± 1.6^bc^		1.81 ± 1.09	> 0.05	0.98 ± 0.44	> 0.05	2.3 ± 1.44^b^		73.76 ± 34.43	> 0.05	2.96 ± 3.78	> 0.05
Cholestatic	5.9 ± 3.76^a^		2.16 ± 1.46		1.11 ± 0.62		3.36 ± 3.02^a^		65.75 ± 31.52		3.48 ± 4.63	
Mix	4.38 ± 1.68^b^		1.88 ± 0.87		0.92 ± 0.46		2.21 ± 1.37^bc^		66.58 ± 29.32		3.35 ± 3.59	
Severity of DILI												
Level 0–2	4.79 ± 1.69	0.12	1.62 ± 0.7	< 0.01	1.22 ± 0.5	< 0.01	2.54 ± 1.02	0.11	62.34 ± 31.89	< 0.01	1.64 ± 1.03	< 0.01
Level ≥ 3	5.63 ± 4.92		2.72 ± 1.2		0.65 ± 0.42		3.28 ± 4.18		82.45 ± 33.29		6.55 ± 5.43	

*TG* triglyceride, *Cho* cholesterol, *LDL-C* low-density lipoprotein cholesterol, *HDL-C* high-density lipoprotein cholesterol, *FFA* free fatty acid, *DILI* drug induced liver injury.

abc: significant difference (P ≤ 0.05) was shown between different letters.

**Figure 3:**
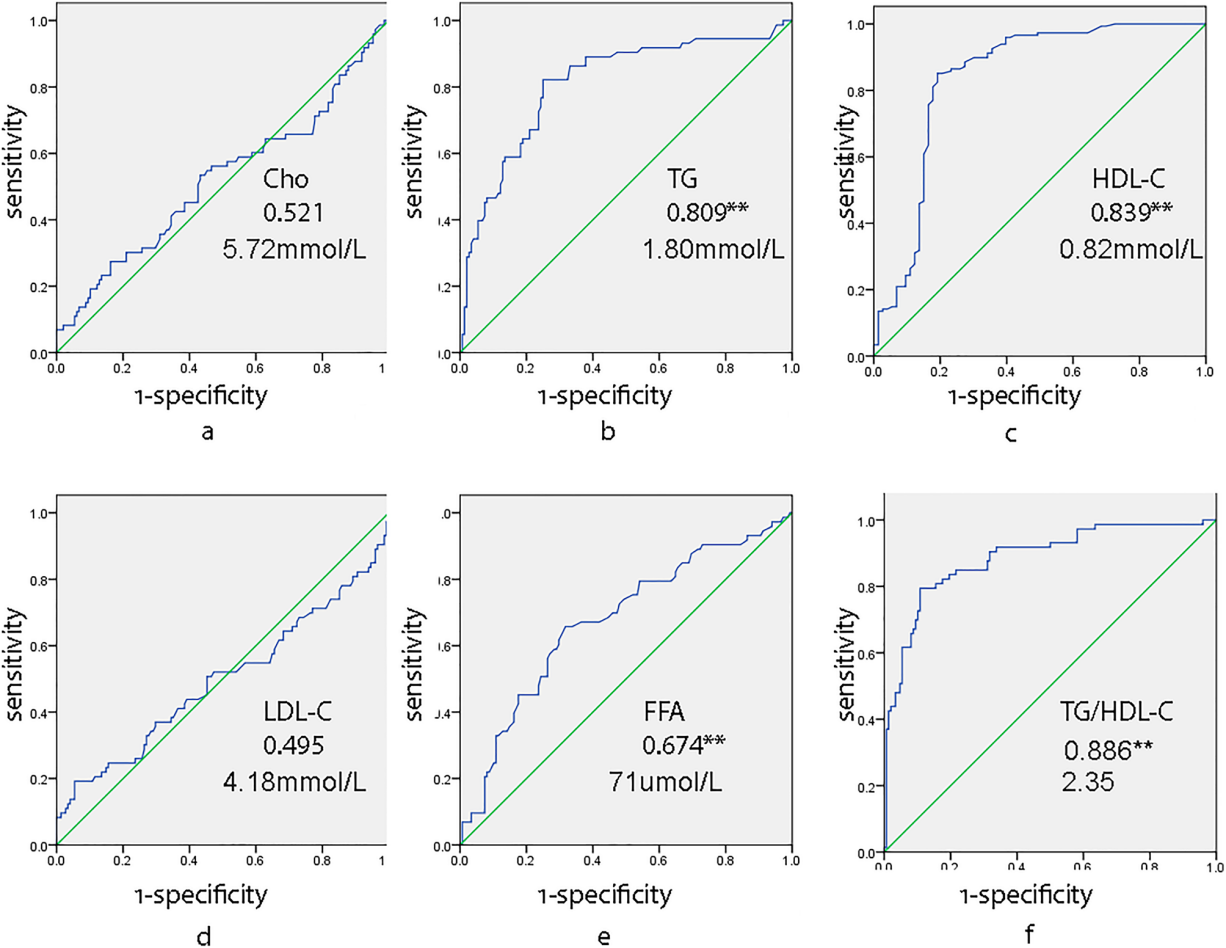
Receiver operating characteristic (ROC) curves of dyslipidemia and TG/HDL-C ratio for diagnosis of severe DILI. *TG* triglyceride, *Cho* cholesterol, *LDL-C* low-density lipoprotein cholesterol, *HDL-C* high-density lipoprotein cholesterol, *FFA* free fatty acid, *DILI* drug induced liver injury. (**a**) AUC and cut-off value of Cho for diagnosis of severe DILI; (**b**) AUC and cut-off value of TG for diagnosis of severe DILI; (**c**) AUC and cut-off value of HDL-C for diagnosis of severe DILI; (**d**) AUC and cut-off value of LDL-C for diagnosis of severe DILI; (**e**) AUC and cut-off value of FFA for diagnosis of severe DILI; (**f**) AUC and cut-off value of TG/HDL-C ratio for diagnosis of severe DILI. **P ≤ 0.01.

DILI patients were divided into mild and severe groups. 98.63% of patients had dyslipidemia in severe group, and 63.51% in mild group (Table 2). Levels of TG and FFA were significantly increased in severe group than those in mild group, and level of HDL-C was significantly lower in severe group than that in mild group. The TG/HDL-C ratio was significantly increased in severe group than that in mild group (Table 4, Fig. 2). To indicate the severity of DILI, AUCs of severe group for TG and HDL were 0.81 (P < 0.05) and 0.84 (P < 0.05) respectively, and the cut-off values were 1.80 mmol/L and 0.82 mmol/L respectively. AUC of severe group for TG/HDL-C ratio was 0.89 (P < 0.05), the cut-off point was 2.35, the youden index was 0.69 (Table 5, Fig. 3). Among liver injury classes, Cho and LDL-C were statistical significantly higher in cholestatic injury group than those in hepatocellular and mixed liver injury groups. Among medication classes of DILI groups, HDL-C was statistical significantly lower in Chinese herbal medicine group than that in western medicine group, TG/HDL-C ratio was significantly higher in Chinese herbal medicine group than western medicine group (Table 4). In mild DILI group, there is no significant difference of serum lipid levels between different medication classes of DILI. In severe DILI group, TG level in combined herbal and western medicine used group was significantly higher than that in western medicine used group. HDL-C level in herbal medicine used group was significantly lower than that in western medicine used group. Above data were shown in Table 6.

**Table 5:** Receiver operating characteristic (ROC) curve analysis of serum lipid levels and TG/HDL-C ratio for diagnosis of severe DILI.

Severity of DILI	Cho	TG	HDL-C	LDL-C	FFA	TG/HDL-C
AUC	0.52	0.81	0.84	0.50	0.67	0.89
Sensitivity	0.27	0.82	0.85	0.19	0.66	0.79
1 − Specificity	0.16	0.25	0.19	0.05	0.32	0.11
Youden index	0.11	0.57	0.66	0.14	0.34	0.69
Cut-off value	5.72 mmol/L	1.80 mmol/L	0.82 mmol/L	4.18 mmol/L	71.00 µmol/L	2.35
p	0.62	< 0.01	< 0.01	0.91	< 0.01	< 0.01

*TG* triglyceride, *Cho* cholesterol, *LDL-C* low-density lipoprotein cholesterol, *HDL-C* high-density lipoprotein cholesterol, *FFA* free fatty acid, *DILI* drug induced liver injury, *AUC* the area under the receiver-operating characteristic curve.

**Table 6:** Dyslipidemia and TG/HDL-C ratio of differnet medication classes in mild and severe DILI.

Severity of DILI	Medication classes	Cho (mmol/L)		TG (mmol/L)		HDL-C (mmol/L)		LDL-C (mmol/L)		FFA (µmol/dL)		TG/HDL-C	
		(Mean ± SD)	P	(Mean ± SD)	P	(Mean ± SD)	P	(Mean ± SD)	P	(Mean ± SD)	P	(Mean ± SD)	P
Level 0–2	Chinese herbal medicines	4.71 ± 1.79	> 0.05	1.54 ± 0.62	> 0.05	1.19 ± 0.44	> 0.05	2.51 ± 1.22	> 0.05	64.81 ± 31.69	> 0.05	1.53 ± 1.19	> 0.05
	Both	4.4 ± 1.61		1.85 ± 0.98		1.16 ± 0.6		2.25 ± 0.66		57.32 ± 22.23		1.92 ± 1.35	
	Western medicines	5.03 ± 1.77		1.64 ± 1.5		1.29 ± 0.46		2.7 ± 1.05		61.28 ± 29.68		1.65 ± 3.03	
Level ≥ 3	Chinese herbal medicines	5.05 ± 4.8	> 0.05	2.75 ± 1.3^ab^		0.57 ± 0.43^b^		2.7 ± 4.02	> 0.05	83.11 ± 31.46	> 0.05	7.17 ± 5.35	> 0.05
	Both	6.14 ± 3.02		3.44 ± 1.35^a^		0.65 ± 0.35^ab^		4.09 ± 3.31		94.9 ± 35.69		7.07 ± 4.81	
	Western medicines	7.04 ± 3.94		2.28 ± 0.97^b^		0.84 ± 0.5^a^		4.45 ± 3.35		72.29 ± 39.2		4.81 ± 5.23	

*TG* triglyceride, *Cho* cholesterol, *LDL-C* low-density lipoprotein cholesterol, *HDL-C* high-density lipoprotein cholesterol, *FFA* free fatty acid, *DILI* drug induced liver injury.

abc: significant difference (P ≤ 0.05) was shown.

### Dyslipidemia and liver function in DILI

**Figure 4:**
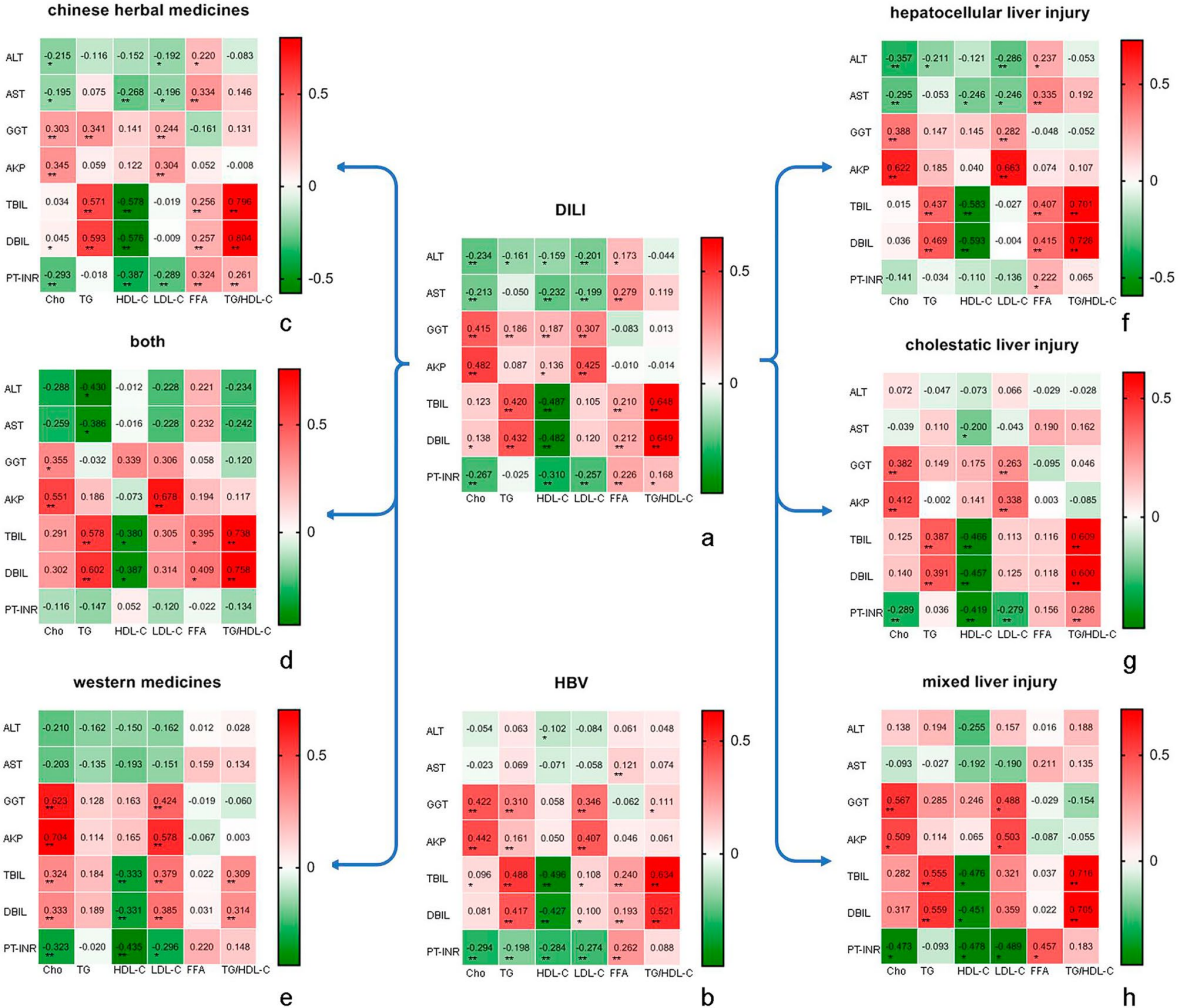
The correlation between liver function and serum lipid levels for different classes of DILI and acute hepatitis B groups. *TG* triglyceride, *Cho* cholesterol, *LDL-C* low-density lipoprotein cholesterol, *HDL-C* high-density lipoprotein cholesterol, *FFA* free fatty acid, *ALT* aminotransferase, *AST* Aspartate transaminase, *GGT* gamma-glutamyl transferase, *AKP* alkaline phosphatase, *TBIL* total bilirubin, *DBIL* direct bilirubin, *PT-INR* International normalized ratio of prothrombin time, *DILI* drug induced liver injury. (**a**) the correlation between liver function and serum lipid levels of DILI patients. (**b**) the correlation between liver function and serum lipid levels of HBV patients. (**c**) the correlation between liver function and serum lipid levels of DILI patients with herbal medicine use. (**d**) the correlation between liver function and serum lipid levels of DILI patients with combined herbal and western medicine use. (**e**) the correlation between liver function and serum lipid levels of DILI patients with western medicine use. (**f**) the correlation between liver function and serum lipid levels of DILI patients with hepatocellular liver injury. (**g**) the correlation between liver function and serum lipid levels of DILI patients with cholestetic liver injury. (**h**) the correlation between liver function and serum lipid levels of DILI patients with mixed liver injury. *P ≤ 0.05, **P ≤ 0.01.

The liver function was compared between DILI group and acute hepatitis B group. Gamma-glutamyl transferase (GGT), alkaline phosphatase (AKP), total bilirubin (TBIL) and direct bilirubin (DBIL) were significantly higher in DILI than those in acute hepatitis B group. International normalized ratio (INR) of prothrombin time (PT) (PT-INR) was significantly lower in DILI than that in acute hepatitis B group. The liver function was also compared between different classes of DILI. Among liver injury classes of DILI, levels of ALT and AST were significantly higher in mixed and hepatocellular liver injury group than those in cholestatic liver injury group. GGT in cholestatic liver injury group was higher than that in hepatocellular liver injury group. AKP in cholestatic and mixed liver injury groups were higher than those in hepatocellular liver injury group. Levels of TBIL and DBIL were higher in mixed group than those in hepatocellular liver injury group. Among differnet medication classes of DILI groups, levels of ALT, AST, TBIL, DBIL and PT-INR in herbal medication group were significantly higher than those in western medication group. Above data were shown in Table 7. The relationship between dyslipidemia and liver function were investigated in DILI and acute hepatitis B groups. The result showed that dyslipidemia was related to liver function. TG/HDL-C ratio was related to TBIL in DILI and acute hepatitis B group, with the correlation coefficient 0.648 and 0.634 (P < 0.01), respectively. In different classes of DILI, TG/HDL-C ratio were related to TBIL and DBIL with significant correlation coefficient. Above data were shown in Fig. 4. Liver functions were compared between different liver injury classes of DILI. Alanine aminotransferase (ALT) and aspartate transaminase (AST) were significantly higher in hepatocellular injury participants and mixed injury participants than those in cholestatic injury participants. GGT level was significantly higher in cholestatic liver injury class than that in hepatocellular injury class. AKP, TBIL and DBIL levels were significantly higher in cholestatic liver injury class and mixed injury class than those in hepatocellular injury class. The result showed that TG/HDL-C ratio was related to TBIL in different classes of DILI, in liver injury classes, the correlation coefficients were 0.701, 0.609 and 0.716 (P < 0.01), respectively; in medication classes, the correlation coefficients were 0.796, 0.738 and 0.309 (P < 0.01), respectively (Fig. 4).

**Table 7:** Liver functions in DILI and acute hepatitis B group.

Variable	DILI	HBV	DILI injury classes			DILI medication classes		
			Hepatocellular liver injury	Cholestatic liver injury	Mixed liver injury	Chinese herbal medicines	Both	Western medicines
ALT(U/L)	384.23 ± 375.12	431.04 ± 631.03	589.52 ± 418.53^ac^	139.21 ± 119.55^b^	653.09 ± 245.58^a^	435.93 ± 381.82^a'^	401 ± 398.75^a'b'^	296.62 ± 341.72^b'^
AST(U/L)	224.05 ± 236.89	253.48 ± 467.91	309.88 ± 275.95^ac^	110.52 ± 113.39^b^	386.57 ± 243^a^	256.81 ± 255.17^a'^	200 ± 192.63^a'b'^	183.53 ± 219.25^b'^
GGT(U/L)	285.24 ± 266.36^##^	154.74 ± 129.09	216.35 ± 201.21^b^	337.48 ± 299.72^a^	330.52 ± 287.1^ab^	260.23 ± 226.6	310.91 ± 231.32	312.99 ± 329.92
AKP (U/L)	181.14 ± 124.27^##^	123.35 ± 52.46	131.95 ± 64^b^	219.34 ± 156.35^a^	209.52 ± 67.72^ac^	163.2 ± 77.2	179.59 ± 111.28	209.7 ± 175.04
TBIL (µmol/L)	93.48 ± 113.49^##^	58.64 ± 76.21	75.87 ± 96.12^b^	100.14 ± 120.97^ab^	135.35 ± 133.68^a^	109.78 ± 118.68^a'^	85.73 ± 94.48^a'b'^	71.5 ± 109.99^b'^
DBIL (µmol/L)	67.54 ± 84.33^##^	42.31 ± 92.03	54.37 ± 71.29^b^	72.35 ± 89.74^ab^	99.64 ± 99.96^a^	78.61 ± 86.17^a'^	62.07 ± 72.22^ab'^	52.71 ± 84.8^b'^
PT-INR	1.08 ± 0.15^##^	1.21 ± 0.26	1.11 ± 0.16	1.05 ± 0.14	1.12 ± 0.15	1.1 ± 0.14^a'^	1.11 ± 0.23^a'b'^	1.05 ± 0.12^b'^

*ALT* aminotransferase, *AST* Aspartate transaminase, *GGT* gamma-glutamyl transferase, *AKP* alkaline phosphatase, *TBIL* total bilirubin, *DBIL* direct bilirubin, *PT-INR* International normalized ratio of prothrombin time.

^#^P ≤ 0.05; ^##^P ≤ 0.01. abc: significant difference (P ≤ 0.05) was shown in different DILI injury classes. a’b’c’: significant difference (P ≤ 0.05) was shown in different DILI medication classes.

## Discussion

Although many drugs are within the range of safe dosage, there is still an increased potential risk for hepatotoxicity. The risk factors of DILI include genetic polymorphisms for xenobiotic-metabolizing enzymes, genetic mitochondrial diseases and pre-existing liver diseases such as NAFLD, alcoholic liver disease, viral hepatitis and liver carcinoma^16^. A retrospective research in Spanish showed that dyslipidemia is a risk factor for chronic liver injury following acute idiosyncratic DILI^17^. However, its mechanism is unclear.

In our study, we have found that dyslipidemia is an independent risk factor of DILI (AOR 4.6; 95% CI 2.81–7.54; P < 0.01) compared to participants without dyslipidemia when compared to medication using group, and compared to acute hepatitis B group (AOR 2.12; 95% CI 1.37–3.29; P < 0.01). The mechanisms are supposed to be the following: First, malnutrition such as high fat diet could decrease medication clearance and lead to slowed elimination and high plasma levels of drugs^16^. Second, the liver and biliary system is an integral part of the reverse cholesterol transport system which prevents accumulation of cholesterol, and liver diseases accompanied with biliary system injury are associated with dyslipidemia^18^.

The relationship between serum lipid and liver injury is still controversial. Previous study has shown that acetaminophen overdose in adult NMRI mice results in temporal homeostasis in hepatic concentrations of free fatty acids and expression of key genes underlying liver lipid metabolism^19^. The results from our study revealed that dyslipidemia is an independent risk factor of severe DILI (AOR 25.31; 95% CI 7.62–83.99; P < 0.01), TG/HDL-C ratio can be used as an indicator of severe DILI (AUC 0.89; p < 0.05). TG/HDL-C ratio was related to TBIL level, with correlation coefficient 0.648 and 0.634 in DILI group and acute hepatitis B group (P < 0.01). As the severity of DILI is directly related to the bilirubin level, the relationship between TG/HDL-C ratio and severe DILI is due to the elevated bilirubin level of severe DILI. Previous studies have showed that cholestatic liver injury is related to dyslipidemia through bile acid-FXR expression^20–22^. Researches showed that liver function in patients with obstructive jaundice accompanied by dyslipidemia was more aggravated than that in patients with simple obstructive jaundice^23^, which is in keeping with our findings. Besides, Cho and LDL-C levels were higher in cholestatic liver injury group than those in hepatocellular and mixed liver injury groups in this study. Cholesterol could synthesize bile acid in liver, liver cholesterol overload could aggravate obstructive cholestasis in mice, and cholestasis resulted in hypercholesterolemia in clinical researches^24–26^.

Our study showed that Chinese herbal medicine is an independent risk factor of DILI and severe DILI. Liver function in herbal medicine group was severe than that in western medicine group in our study, which may result from the increasing prevalence of Herbal medicine as complementary and alternative medicine therapies^27^. It was observed that more than 18.6% of the adult population in America used herbs for the benefit of their health^28,29^. The World Health Organization has estimated that 80% of people worldwide rely on herbal medicines for part of their primary health care needs^30^. In our research, 66.06% (50.68% + 15.38%) of DILI patients used herbal medicines and 28.45% (5.69% + 22.76%) of medication control participants in hospital used herbal medicines. Recent studies have reported a large amount of herbal induced liver injury^31^. It has been shown that herb-induced liver injury is associated with a higher frequency of hepatocellular injury and cholestatic liver injury in females^32,33^. Herbal products need to be carefully separated into multiple components and toxicological studies should be conducted to determine their adverse effects. Previous studies have demonstrated that DILI occurs more frequently in females^34,35^. Our research found that males had a lower risk of DILI compared with females when compared with medication using control group and liver injury control group, which is in keeping with previous findings.

## Conclusion

Our study confirmed the relationship between TG/HDL-C ratio and the severity of DILI, and the association between TG/HDL-C and TBIL level in liver injury diseases. This feasible index could be applied to better treatment of DILI in clinical practice. The results also implied that the effects of dyslipidemia should be evaluated after DILI regression. The mechanism of dyslipidemia in DILI needs further investigation.

## Limitations

This was a single-centered retrospective study, which relied on the accuracy of medical records. This research only applies to untreated patients with dyslipidemia, but patients with DILI caused by lipid-lowering drugs were not analyzed. There was also limited available data, for instance, the body mass index (BMI) and bile acid in the patient population. The relationships between BMI, bile acid and DILI are unclear. Animal and molecular biology researches need to be investigated.

In our research, hospitalized participants who had long-term medication use were enrolled in the medication using group. However, information of outpatients with long-term medication especially herbal medicine use was incomplete and difficult to obtain. There is a consistent disproportion significant difference of drug indications between DILI cases and controls, even if we have corrected for drug exposures, the results from the comparison with medication control group are possibly due to the underlying medical conditions associated with the selection of the controls. The medication using group size should increase and outpatients’ information should be collected in future studies.

## Materials and method

In this single-centered observational study, we retrospectively collected the information of participants from Jan. 1, 2017 to Dec. 31, 2021 from Qilu Hospital of Shandong University. The Independent Institutional Review Board of Qilu Hospital approved the study protocol (No. KYLL-202008-097). All the clinical information was directly from hospital electronic medical records. Since all the data are anonymous, the informed consent waiver statement was approved by the ethics committee of Qilu Hospital. All methods were performed according to the relevant regulations and guidelines.

Sample size was calculated by the corresponding formula using PASS version 11.0 for windows. The ratio of dyslipidemia in control group was 34.4% in our research, and the expected odd ratio was 2.173 in a recent study^7^ (α = 0.05, β = 0.2). After calculation, the sample size of DILI group and the control group were at least 108.

### Study population

A study of all medical registries with International Classification of Disease-10(ICD-10) diagnoses related to DILI was performed from Jan. 2017 to Dec. 2021 in Qilu hospital, and exclusion criteria were as follows:

1. Incomplete serological test results.
2. Participants with known pre-existing liver injuries, including viral hepatitis, autoimmune liver disease, and liver cancer according to medical records.
3. Use of lipid-lowering drugs including fibrates, statin or herbal medicines.
4. RUCAM score < 6.
5. Malignant tumor patients with multiorgan metastasis.
6. Under 18 years of age.

Participants with chronic diseases like rheumatoid arthritis, ankylosing spondylitis, hypertensions or diabetes who had long-term medication use were enrolled in the medication using group. Medication used especially western medicine in control group had liver toxicity in the pharmaceutical directions. Exclusion criteria include less than 18 years old, using fibrates or statins, pre-existing liver diseases, pregnancy, malignant diseases with multiorgan metastases, or organ failure.

We couldn’t judge which medication is responsible of DILI in patients with concomitant medications, while we kept a record of all medications prescribed for each DILI cases. Medications were classified into Chinese herbal medicines, western medicines and both. Medications were also categorized according to drug indications in our research.

Participants with acute hepatitis B were enrolled in liver injury control group. Information of medical registries with International Classification of Disease-10(ICD-10) diagnoses related to acute hepatitis B was collected from Jan. 2017 to Dec. 2021 in Qilu hospital. The diagnosis of acute hepatitis B was defined as positive for HBsAg, the discrete onset of symptoms like fever, headache, malaise, anorexia, nausea, vomiting, diarrhea and abdominal pain, the presence of jaundice or elevated alanine aminotransferase (ALT) level^36^. The exclusion criteria include: less than 18, using fibrates or statins, pregnancy, malignant diseases with multiorgan metastases and organ failure.

### Diagnoses of DILI

Roussel Uclaf Causality Assessment Method (RUCAM) is a scoring system to quantify the association between liver injury and implicated medicines^37,38^. In our study, all the patients diagnosed with DILI were assessed by RUCAM scale. A final summary score allows categorization of causality as excluded (< 0), unlikely (1–2), possible (3–5), probable (6–8), or highly probable (> 8). The study excluded patients with RUCAM score less than 6.

### Diagnosis of dyslipidemia

Patients were considered to be with dyslipidemia if they had Cho > 6.00 mmol/L or 240 mg/dL, TG > 1.7 mmol/L or 200 mg/dL, HDL-C < 0.8 mmol/L or 40 mg/dL, or LDL-C > 3.37 mmol/L or 160 mg/dL.

### Severity of DILI

The severity of DILI was identified as different levels according to Chinese Society of Hepatology Guidelines. Clinical and laboratory data were collected to calculate the severity score. DILI severities were classified as mild (level 1) for alkaline phosphatase (ALP) and/or serum ALT elevations in the absence of jaundice, with total bilirubin (TBil) < 2.5 ULN; moderate (level 2) by increased ALT and/or ALP, jaundice with TBil ≥ 2.5 ULN, or coagulopathy, with international normalized ratio (INR) ≥ 1.5; moderately severe (level 3) by increased jaundice with TBil ≥ 5 ULN or INR ≥ 1.5; severe (level 4) by jaundice with TBil ≥ 10 ULN or TBil elevation ≥ 17.1umol/L per day, INR ≥ 1.5 or prothrombin activity (PTA) < 40% and liver or other organ failure; and fatal (level 5) by death from liver disease or the need for liver transplantation surgery^15^.

### Classification of DILI

First, DILI patients were divided into mild group (level 0–2) and severe group (≥ level 3) according to the severity of DILI. Second, DILI patients were divided into hepatocellular injury group (R ≥ 5), mixed liver injury group (2 < R < 5) and cholestatic liver injury group (R ≤ 2) (R is ALT to ALP ratio). Third, DILI patients were divided into Chinese herbal medicine-induced group, western medicine-induced group and combination-induced group according to the drug classes of DILI. Indications of drugs in DILI and medication using group were evaluated.

### Demographic and clinical variables

The demographic characteristics were evaluated in this study, including sex, age, smoking history, alcoholic drinking history, hypersensitivity, cardiovascular disease (CVD), hypertension, malignancy, type 2 diabetes, and family chronic diseases. Data about the cause of liver damage, patterns of liver disease, and medications that cause DILI in patients were analyzed.

Biochemical parameters from routine laboratory tests of the patients were also evaluated at the time of admission in hospital, including ALT, AST, GGT, ALP, TBIL, DBIL and PT-INR. Serum lipid levels, including Cho, TG, HDL-C, LDL-C, and FFA were collected.

### Statistic analysis

Percentages were used for qualitative parameters. Differences in proportions between groups were evaluated using χ^2^-test. Mean ± standard deviation (mean ± SD) was used for normally distributed continuous variables. Correlation coefficients were obtained using Pearson’s correlation. Bi-variate logistic regressions were used to investigate the relationship between variables. We used univariate analysis to select variables with P < 0.1 to adjust for potential confounding effects, and multivariate logistic regression analyses were performed, including adjusted odds ratios (AORs) and 95% confidence intervals (CIs). The area under the receiver-operating characteristic curve (AUC) was used to validate the effectiveness of the variable and to define optimal cut-off values. Data were examined graphically and numerically. P value less than 0.05 was considered statistically significant. SPSS version 17.0 (SPSS, Chicago, IL) and GraphPad Prism 8.0 for Windows were used to perform statistical analysis.

## Data Availability

All data generated or analyzed during this study are available from the corresponding author upon request.

## Abbreviations

DILI: Drug-induced liver injury
RUCAM: Roussel Uclaf causality assessment method
TG: Triglyceride
Cho: Cholesterol
HDL-C: High-density lipoprotein cholesterol
LDL-C: Low-density lipoprotein cholesterol
FFA: Free fatty acid
ALT: Alanine amino transferase
AST: Aspartate transaminase
GGT: Gamma-glutamyl transferase
AKP: Alkaline phosphatase
TBIL: Total bilirubin
DBIL: Direct bilirubin
PT-INR: International normalized ratio of prothrombin time
AUC: An area under the curve
